# Age, chronic non-communicable disease and choice of traditional Chinese and western medicine outpatient services in a Chinese population

**DOI:** 10.1186/1472-6963-9-207

**Published:** 2009-11-17

**Authors:** Vincent CH Chung, Chun Hong Lau, Eng Kiong Yeoh, Sian Meryl Griffiths

**Affiliations:** 1 School of Public Health and Primary Care, Chinese University of Hong Kong, Hong Kong SAR, PR China

## Abstract

**Background:**

In 1997 Hong Kong reunified with China and the development of traditional Chinese medicine (TCM) started with this change in national identity. However, the two latest discussion papers on Hong Kong's healthcare reform have failed to mention the role of TCM in primary healthcare, despite TCM's public popularity and its potential in tackling the chronic non-communicable disease (NCD) challenge in the ageing population. This study aims to describe the interrelationship between age, non-communicable disease (NCD) status, and the choice of TCM and western medicine (WM) services in the Hong Kong population.

**Methods:**

This study is a secondary analysis of the Thematic Household Survey (THS) 2005 dataset. The THS is a Hong Kong population representative face to face survey was conducted by the Hong Kong Administrative Region Government of China. A random sample of respondents aged >15 years were invited to report their use of TCM and WM in the past year, together with other health and demographic information. A total of 33,263 persons were interviewed (response rate 79.2%).

**Results:**

Amongst those who received outpatient services in the past year (n = 18,087), 80.23% only visited WM doctors, 3.17% consulted TCM practitioners solely, and 16.60% used both type of services (double consulters). Compared to those who only consulted WM doctor, multinomial logistic regression showed that double consulters were more likely to be older, female, NCD patients, and have higher socioeconomic backgrounds. Further analysis showed that the association between age and double consulting was curvilinear (inverted U shaped) regardless of NCD status. Middle aged (45-60 years) NCD patients, and the NCD free "young old" group (60-75 years) were most likely to double consult. On the other hand, the relationship between age and use of TCM as an alternative to WM was linear regardless of NCD status. The NCD free segment of the population was more inclined to use TCM alone as they become older.

**Conclusion:**

In Hong Kong, most patients have chosen WM provided in the public sector as their sole outpatient service provider for NCD. Amongst TCM service users, middle aged NCD patients are more likely to choose both TCM and WM outpatient services. Meanwhile, older people without NCD are more likely to use TCM as their main form of care, but the size of this population group is small. These utilization patterns show that patients choose both modalities to manage their NCD and TCM should be considered within policies for supporting patients with NCD under the wider primary health and social care system that supports patient choice.

## Background

In contemporary Chinese health culture, the concept of complementarity between traditional Chinese medicine (TCM) and western medicine (WM) is well received in the community. TCM and WM are thought to possess respective strengths and weaknesses: TCM is considered to be slow in action but more thorough in "curing the root of the problem", while western medicine is "more powerful and quick" but may also cause significant side effects [1]. The preference for using both TCM and WM may stem from perceptions that synergism between the two would enhance clinical improvement, with each modality addressing different aspects of the illness. However, TCM may be chosen as an alternative to WM when patients perceive the need for health maintenance or tonic care [2,3]. This long established simultaneous use of both TCM and WM by the Chinese population has shaped China's modern health policy ---- the coexistence and integration of TCM and WM at all levels of healthcare has been a salient feature of the Chinese healthcare system since the establishment of the People's Republic of China [4]. Today, as its affluence grows, China's population is ageing and the burden on non-communicable diseases (NCD) is growing. In response to this challenge, the Chinese healthcare system is currently under reform [5] and whilst TCM is recognised as an important part of the healthcare system but its contribution to the control of NCD has yet to be fully articulated [6].

Hong Kong has been a Special Administrative Region (SAR) of China since 1997 when the British colonial rule ended. As required by the new constitutional law [7], TCM gained official recognition within the Hong Kong healthcare system and subsequently formal regulation was introduced in 1999 [8]. Currently, WM and TCM co-exist in Hong Kong's healthcare system within public and private sectors. About 30% of all outpatient episodes are managed by the tax funded public sector, which predominantly provides WM services at a minimal out of pocket fee. The fees in public sector TCM clinics are substantially higher as they are being run on a self-financed basis [8]. The remaining 70% of outpatient care is provided by WM or TCM clinicians in the private sector, financed either by out of pocket household expenditure, or to a lesser extent, by private insurance schemes. The majority of these schemes belongs to indemnity policies which pay clinicians on a fee for services basis, and allow patient reimbursement with a cap on the maximum claim allowed. The remainders are capitation policies which resemble various forms of managed care, including contract medicine, prepaid plans and preferred provider networks. In 2002, the TCM sector was providing 19% of all outpatient care in Hong Kong [9].

Revitalization of primary healthcare (PHC) is high on the healthcare reform agenda in both developing and developed countries [10]. People centeredness is the foundation of an effective and efficient PHC system [11], in which critical elements include person centeredness, comprehensiveness and integration, continuity of care, and participation of patients, families and communities [12,13]. Under the principal of people centeredness, TCM should presumably be included as an integral part of the Hong Kong PHC system and it could be expected that TCM treatment would be orchestrated with other PHC services. However, unlike the inclusive policy adopted in mainland China where many clinics provide both modalities of care, Hong Kong's TCM practice is still operating in parallel to the dominant WM sector as well as the wider PHC professional community [14]. Although the Hong Kong Chief Executive first announced TCM development in 1997 [15] proposals for effective coordination of TCM and WM care have not emerged from the government. Further top down commitment to policy change appeared in the current Chief Executive's election manifesto in 2008, explicitly calling for the "integration of professional Chinese medicine practice into the public healthcare system" and "development of traditional Chinese medicine" as part of the future health care reform in Hong Kong [16]. However the latest two healthcare reform discussion papers "Building a Healthy Tomorrow [17]" and "Your Health Your Life [18]" fail to mention the role of TCM in PHC. Discussion on the future development of integration between TCM and WM, and how to position the TCM sector for addressing the health system's strategic goals have failed to reach the healthcare reform agenda, despite TCM's public popularity and its potential in tackling the NCD challenge [19]. Such issues are also highly relevant to policy makers in other ageing societies where popularity of complementary and alternative medicine (CAM) is increasing [20]. This paper explores the choice of TCM in management of NCD by analysing outpatient utilisation in a large population sample within the SAR.,

## Methods

### Data collection

We conducted secondary data analysis using the dataset of Thematic Household Survey (THS) 2005 coordinated and managed by the Census and Statistic Department (CSD) of the Hong Kong SAR Government. In the conduct of all survey exercise, the CSD complies with the Declaration of Professional Ethics of the International Statistical Institute [21]. Official approval for data utilization was obtained. THS 2005 is a population representative, cross-sectional survey conducted between November 2005 and March 2006. All household samples were approached for face to face interviews during personal visits by trained enumerators. The THS covered the entire land-based, non-institutional population of Hong Kong. A total of 33,263 non-institutional (response rate: 79.2%) persons were interviewed. All sample data were weighted to achieve population representation using standard census methodologies by the CSD. The total sample represented 6,750,652 persons of the local general population. Questions on use of TCM (Chinese herbalism, acupuncture and therapeutic massage) and WM outpatient services in the past 12 months, self reported NCD status as informed by a WM doctor (including stroke, epilepsy, Parkinson's disease, high blood pressure, heart failure, lung disease, thyroid disease, diabetes mellitus, liver disease, kidney disease, stomach and intestinal disease, musculoskeletal disease, immune disease, skin disease, depression, and anxiety disorder), and demographic data including age, gender, education level, monthly personal income and possession of medical insurance were asked among respondents aged 15 or above [22].

### Statistical analysis

The use of TCM or WM outpatient services in the past year were treated as dependent variables (1. visited TCM practitioners only, 2. visited WM doctors only, or 3. consulted both types of clinicians - double consulters). Other demographic and health related factors were regarded as independent variables and they were entered simultaneously into a multinomial logistic regression model such that their effects on the choice for TCM, WM or both were evaluated. Specifically, quadratic age term was used to test for a curvilinear effect of age, and the two-way interaction effect between age and NCD status was also assessed. The probabilities of using each type of services were calculated from the regression model, plotted across the age spectrum, and were further stratified according to NCD status. Statistical tests were two-sided with significant level of 0.05. SPSS 14.0 (SPSS Inc., Chicago, IL, USA) was used. All analyses were performed using the weighted dataset.

## Results

Amongst those who received outpatient services in the past year (n = 18,087), 80.23% visited WM doctors while 3.17% consulted TCM practitioners only. 16.60% used both type of services. When compared to sole WM users, this group of double consulters had a significantly higher usage of WM service (mean yearly frequency for double consulters: 7.47, 95%CI: 7.20,7.74; for sole WM users: 5.19, 95%CI: 5.10,5.27). However, the mean frequency of TCM visits by double consulters was not significantly different from those who only consulted TCM practitioners (mean yearly frequency for double consulters: 7.16, 95%CI: 6.82,7.51; for sole TCM users: 6.45, 95%CI: 5.72,7.19). Other demographic and healthcare related characteristics are shown in Table 1.

**Table 1:** Demographic and health-related factors of respondents

		**%**
**Gender**	Male	47.0
	Female	53.0
**Education**	Primary or below	27.6
	Secondary	54.0
	Tertiary	18.4
**Possession of WM insurance**	No	74.6
	Yes	25.4
**Possession of TCM insurance**	No	92.9
	Yes	7.1
**Age group**	15-19	7.3
	20-29	14.5
	30-39	18.3
	40-49	22.8
	50-59	16.8
	60-69	8.9
	70-79	8.0
	80 or above	3.5
**Self reported NCD status**	No	77.1
	Yes	22.9
**Personal monthly income (US$1 = HK$7.8)**	HK$0-3999	39.5
	HK$4000-7999	16.8
	HK$8000-12499	19.4
	HK$12499 or above	24.2

Key: NCD = non-communicable disease, WM = western medicine, TCM = traditional Chinese medicine

Compared to those who only consulted WM doctor, multinomial logistic regression showed that sole TCM users were more likely to be older, female and have received tertiary education. These three characteristics were also more likely to be observed amongst double consulters, in addition to their significantly higher NCD prevalence and monthly personal income. However, when the interaction effect between age and NCD status was considered, we observed a significant negative association between being an older NCD patient and use of TCM, either solely or in conjunction with WM. The quadratic age term is also found to be significantly associated with double consulting that indicates a curvilinear relationship between age and choice for both WM and TCM (Table 2).

**Table 2:** Multinomial logistic regression: Demographic factors, healthcare-related factors, and use of TCM as a complement or alternative to WM

		Consulted TCM practitioners only	Consulted both TCM practitioners and WM doctors
**Gender**	Female	1.221 (1.028, 1.450)*	1.742 (1.603, 1.893)***
	Primary or below (ref.)	1.00	1.00
**Education**	Secondary	1.043 (0.833, 1.307)	0.927 (0.832, 1.033)
	Tertiary	1.352 (0.987, 1.852)	1.009 (0.867, 1.176)
**Personal monthly income**	With each increment of HK$1000	0.997 (0.988, 1.005)	1.007 (1.003, 1.010)***
**Insurance**	With WM insurance	0.784 (0.624, 0.985)*	0.948 (0.852, 1.054)
	With TCM insurance	1.165 (0.806, 1.684)	2.029 (1.750, 2.351)***
**Age**	With increment of 1 year	1.057 (1.030, 1.085)***	1.072 (1.058, 1.086)***
**Age^2^**		1.000 (0.999, 1.000)*	0.999 (0.999, 1.000)***
**Self reported NCD status**	Yes	2.102 (0.756, 5.840)	1.797 (1.244, 2.597)**
NCD * Age interaction		0.961 (0.944, 0.978)***	0.992 (0.985, 0.998)*

*p < 0.05 **p < 0.01 ***p < 0.001

Key: NCD = non-communicable disease, WM = western medicine, TCM = traditional Chinese medicine

**Figure 1:**
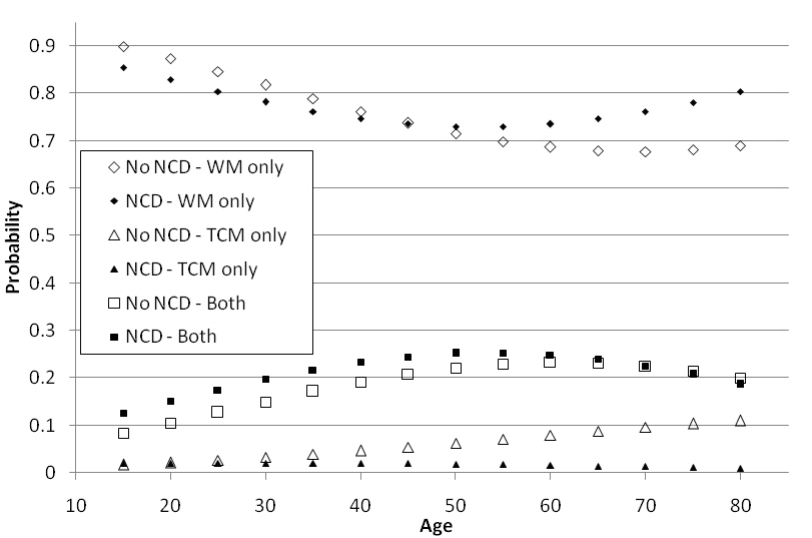
Age differences in the use of TCM as a complement or alternative to WM by NCD status*. Key: NCD = non-communicable disease, WM = western medicine, TCM = traditional Chinese medicine. *Estimated probability when other variables in the regression are kept constant (i.e. female, secondary education, monthly income $HKD 10000, no WM and TCM insurance.

We explored the interrelationship between age, NCD status and service use further graphically. In figure 1, Y-axis indicated the probability of using WM, TCM or both in the past year while age was plotted on X-axis. We held other demographic factors constant by assuming that the individual represented on the graph to be female, secondary educated, having a monthly personal income of HKD$ 10000 (i.e. median monthly income of the Hong Kong population, approx USD$: 1282). At all ages, probability of visiting WM doctor only was the highest, followed by double consulting and using TCM exclusively. Among those who had NCD, the probability of using TCM only remained low and decreased gradually as age advanced. However, the opposite trend was observed among those who were free from NCDs: the probability of only consulting TCM practitioners increased with age. Regardless of NCD status, the association between age and double consulting was curvilinear (inverted U shaped). This curvilinear pattern was also observed in the relationship between age and consulting WM doctors only, but the curve was found to be U shaped. Hence, the curves for double consulting and using WM only formed two hyperbolas: with the presence of NCD, vertex of the hyperbola was located at 45-60 years; while for those who were free from NCD, the vertex appeared at the range of 60-75.

## Discussion

### Main finding of this study

Our results show that the majority of Hong Kong patients have chosen WM doctors as their sole outpatient service providers. Amongst those who used TCM outpatient services, most of them tended to choose between TCM and WM rather than use TCM as an exclusive form of care. Regardless of NCD status, the relationship between age and double consulting was curvilinear (inverted U shaped). Middle aged (45-60 years) NCD patients, and the relatively healthy "young old" group (60-75 years) were most likely to double consult. On the other hand, the relationship between age and using TCM only in the past year was linear regardless of NCD status. The NCD free segment of the population was more inclined to use TCM alone as they became older.

### What is already known on this topic

Studies on the relationship between age, NCD status and CAM use have been increasing over the past decade. In Asia, several Taiwanese studies with national coverage concluded that CAM/TCM use peaked amongst the middle aged [23,24], and self reported NCD status was linked to higher chance in using CAM/TCM [25]. In Singapore, a public sector WM clinic based survey reported that the use of CAM amongst the older population was related to presence of NCD [26], and in Tokyo, Japan, CAM use was associated with multi-morbidity but the effect of age was insignificant, as suggested by a cross-sectional study performed in primary and secondary care clinics [27]. National survey conducted in the US suggested that the relationship between age and CAM use was curvilinear [28]. Adults with NCD [29] or more self-reported unhealthy days [30] were more likely to use CAM, as reported by a US wide study and a regional study conducted in Minnesota respectively. In Canada, a territory wide survey reported that middle-aged, multi-morbid patients were more likely to be frequent CAM users [31], and regional studies suggested that they tended to use WM services heavily [32,33]. Similar patterns emerged in Europe, in which British CAM users were more likely to be middle-aged NCD patients with lower quality of life [34,35]. German CAM users in West Pomerania tended to report lower self rated physical health and multi-morbidity statuses, and were utilizing more WM outpatient services [36]. Australia is an exception to this pattern as reported by a national survey, where the use of CAM tended to decline with age, and CAM users' self-reported health status was not significantly different from non users [37]. This echoes with another study conducted in southern Australia, in which CAM consultation was motivated by desire for counseling for general health issues or for maintenance of health and fitness [38].

### What this study adds

To our knowledge, this study is one of the first population based surveys which illustrates the relationship between TCM use, age and NCD status in the Hong Kong Chinese population. Our finding of middle-aged NCD patients shows a preference for using both TCM and WM that is concordant with other studies described above. For this utilization pattern, we can hypothesise that TCM may be regarded as a therapeutic complement to WM in coping NCD. Further research is needed to clarify how TCM is used alongside WM: whether NCD patients were using both TCM and WM for treating their NCD, or they were choosing TCM and WM for different aspects of their health maintenance. In addition, our result suggested that TCM is used as an exclusive form of care by a small segment of the population, particularly those who are aging and NCD free. The role of TCM in prevention, facilitation of healthy aging and referral to WM needs further exploration.

### Policy implications

If our results are placed within the current policy context of Hong Kong, several questions arise. Firstly, since NCD is a growing health burden for Hong Kong's elderly population TCM needs to be considered in the policy discussion of the recently published NCD. The choice of patients with NCD to seek care from TCM practitioners as well as to consult western doctors may be motivated by the complexity of their conditions, which in turn reflects a significant, long term health burden [39]. This in turn has implications for both healthcare providers and policy makers. Current barriers between TCM and WM professionals exist [8] partly due to institutional arrangements which set the professions up in parallel and partly to do with education and lack of cross fertilisation of ideas and experience. Mutual learning could allow a better understanding on the motivations and expectations of NCD patients who choose to double consult [40]. This could in turn encourage patients to disclose their TCM/WM use to both types of healthcare providers thus preventing potential herbal drug interaction which is known to be a problem [3]. A better knowledge of the evidence base of TCM and WM could facilitate referrals between the two professional groups, especially in the management of NCD where WM has little to offer while TCM could improve outcomes (e.g. Chinese herbal medicine for irritable bowel syndrome [41], acupuncture for osteoarthritis of the knee [42]). Equipped with this knowledge, TCM and WM professionals could therefore assist their patients in making evidence-based choice between TCM and WM.

Another key policy concern is the structure of primary care and the role of TCM within it. A PHC system featuring continuity, coordination and comprehensiveness that includes both WM and TCM could potentially enhance the quality and efficiency of chronic care [43]. However, the current compartmentization of TCM and WM inhibits NCD patients from transiting seamlessly between the two modalities, and subsequently hinders the potential synergy. Despite the presence of paradigm differences between the two modalities of medicine [44], a policy initiative bringing WM and TCM together under a common primary healthcare infrastructure would enhance both patient choice and their well-being. This would involve an alignment not only of practitioners but of statutory organisations. Currently, the professional standards for WM and TCM are set separately by two statutory councils. Joint working between the two regulatory bodies is needed to clarify the ethical, legal and professionals barriers that hinder inter-professional collaboration. Mutual recognition of practice would need to be established to overcome barriers such as the inability to formally refer and share patient information [45]. A further problem is the fragmented nature of both WM and TCM primary healthcare services which present structural obstacles for collaboration in the care of patients with NCD [46]. In Hong Kong, both TCM and WM outpatient services are dominated by the private sector and there is no infrastructure to facilitate collaboration or information exchange within the PHC system. The proposed establishment of a universal electronic patient record system would be a potential solution to bring various healthcare services sectors (primary, secondary and tertiary care, private and public sector, TCM and WM) under a single care coordination platform for common disease such as diabetes and hypertension [47].

A further consideration is how the TCM sector can facilitate active ageing in older but relatively healthy people within the population, and contribute to the maintenance of their independence and addressing risk factors of NCD [48]. The promotion of active ageing is proposed by the WHO as a major policy solution for managing the challenges of ageing population worldwide. Active ageing is defined as the process of optimizing opportunities for health, participation and security in order to enhance quality of life as people age [49]. Our results showed a positive relationship between age and use of TCM amongst those who are free from NCD. To take full advantage of this opportunity, TCM doctors need to be part of the government's strategic plan for NCD and supported to deliver health promotion, disease management, care coordination and social service referrals along with traditional tonics and therapy. However, the preparedness of the Hong Kong TCM practitioners to provide such a service is uncertain [50].

### Strengths and limitations of this study

One of the most notable strengths of this study is that we avoided selection bias by drawing a random sample from Hong Kong that is representative to the Chinese population of the territory whose origin is similar to the population of Southern China. Meanwhile, our study has a number of limitations. First, the cross-sectional nature of our survey did not allow any conclusion on causality. A longitudinal design is needed to ascertain whether the associations observed between various background factors and healthcare choices are causal. Second, respondents were asked to recall the outpatient service utilization over the preceding year and thus recall bias could have led to inflation or deflation of visit frequencies. Finally, the quantitative nature of our study has prevented us from gaining a deeper insight on respondents' motivations and beliefs. Further inquiry using a qualitative approach is warranted.

## Conclusion

The prevalent use of both TCM and WM in the population suggests that both modalities constitute part of the primary healthcare system in Hong Kong. For those with NCD, TCM could take a role in maintaining health and for others without diseases; TCM use could offer an opportunity for promoting health and well-being. The TCM sector could be an important partner with WM in tackling the challenge of increasing NCD burden, as well as a key player in promoting active ageing. In order to explore the potential synergistic benefit of integrated TCM-WM care, further researches on how TCM may be included within policies for supporting patients with NCD is warranted.

## Competing interests

The authors declare that they have no competing interests.

## Authors' contributions

VC, LCH and SG conceived the research idea. LCH conducted the statistical analysis. VC interpreted the result and wrote the first draft of the manuscript. SG and EKY added critical comments on the interpretations of data and on the manuscript. SG and EKY supervised the whole research process. All authors read and approved the final manuscript.

## Pre-publication history

The pre-publication history for this paper can be accessed here:

http://www.biomedcentral.com/1472-6963/9/207/prepub
